# The homologous lipocalins Can f 1 and Lcn-1 induce different immune responses

**DOI:** 10.1186/2045-7022-4-S2-O13

**Published:** 2014-03-17

**Authors:** Beate Posch, Christian Irsara, Martin Herrmann, Dietmar Fuchs, Norbert Reider, Bernhard Redl, Christine Heufler

**Affiliations:** 1 Medical University of Innsbruck, Department of Dermatology, Innsbruck, Austria; 2 Medical University of Innsbruck, Department of Anaesthesiology, Innsbruck, Austria; 3 Medical University of Innsbruck, Division of Medical Biochemistry, Biocenter, Innsbruck, Austria; 4 Medical University of Innsbruck, Division of Molecular Biology, Biocenter, Innsbruck, Austria

## Background

Why and when the immune system provokes TH2 mediated allergic immune responses is still not well understood. Dendritic cells are one of the master regulators during the induction of allergic airway inflammation providing stimuli for TH2 cell differentiation. Most of the major as well as minor mammalian allergens causing respiratory sensitization belong to the lipocalin family. We investigated two homologous lipocalins, the endogenous non-allergenic human Lcn-1 and the major respiratory dog allergen Can f 1 to study their effects on human monocyte derived dendritic cells in order to initiate allergic immune responses.

## Methods

We measured factors involved in directing the type of immune responses including antigen uptake, maturation induction, tryptophan breakdown and cytokine production by human monocyte derived dendritic cells and characterized the type of immune response induced in mixed leukocyte reactions by key cytokine secretion (IFNγ for TH1, IL13 for TH2) or key transcription factor expression (FoxP3 for regulatory T cells) in T cells.

## Results

We found that the homologous lipocalins had differential effects on dendritic cells depending on their allergenic potential. The dog allergen Can f 1 induced less maturation marker expression, less tryptophan breakdown and less IL12 production in human monocyte derived dendritic cells when compared to the endogenous non-allergenic Lcn-1. As a consequence, T cells stimulated by dendritic cells treated with Can f 1 produced more of the TH2 signature cytokine IL13 and less of the TH1 signature cytokine IFNγ than T cells stimulated by Lcn-1 treated dendritic cells.

## Conclusion

The major respiratory dog allergen Can f 1 induces changes in human monocyte derived dendritic cells leading to a TH2 immune response. In contrast, the impact of the highly homologous human Lcn-1 on the dendritic cells induces TH1 cell differentiation. These data indicate that the crosstalk of dendritic cells with lipocalins has the potential to direct the type of the immune response. Our data contribute to the field by showing that human monocyte derived dendritic cells orchestrate immune responses in responding differentially to two highly homologous lipocalins according to their allergenic potential.

